# Change in Land Use and Evapotranspiration in the Manas River Basin, China with Long-term Water-saving Measures

**DOI:** 10.1038/s41598-017-18030-5

**Published:** 2017-12-19

**Authors:** Guang Yang, Lianqing Xue, Xinlin He, Cui Wang, Aihua Long

**Affiliations:** 1 College of Water and Architectural Engineering, Shihezi University, Shihezi, China; 2 Xinjiang Production and Construction Group Key Laboratory of Modern Water-Saving Irrigation, Xinjiang, China; 3 Hydrology and water resources College, Hohai University, Nanjing, China

## Abstract

Widespread application of water-saving measures, especially advanced drip irrigation technologies, may significantly impact on the land use, and further potentially alter regional ecological environments in an arid area. In this study, the remote sensing and geographic information system technology were used to analyze the LANDSAT images (1976–2015) and the MOD16 evapotranspiration data (2000–2015) in the Manas River Basin (MRB), China where the water-saving technologies have experienced the past 40 years. Our results show that the area of the cultivated land was approximately doubled from 1976 to 2015 with a dynamic degree of cultivated land ranging from 1.7% to 4%. The reclamation rates were estimated at 9.5% in 1976 and 21.8% in 2015 and the comprehensive index of land use degree shows an increasing trend in the MRB. The evapotranspiration in the MRB suggests that the cultivated land is becoming more humid while the other regions are becoming more arid. Long-term change in the land use is mainly promoted due to the multiple years’ efforts on development of the water-saving technologies. This study greatly improves our understanding of the interactions between change in ecological environments and human activities and may provide policy makers guidance of sustainable development at an arid area.

## Introduction

With the continuous economic development of societies, more pressure has been placed on global fresh water resources^1–7^. Especially in the last century, net arable lands show a significant expansion driven by economic growths, further worsening shortage in water resources at various scales^8–10^. To address shortage in water resource and uneven distribution of water resources, water-saving measures are necessary to improve usage efficiency of water resource, such as dams and reservoirs, drip irrigation, water channel anti-seepage and other techniques^11^. These water conservation measures are typically used in local areas, and especially in arid and semi-arid areas, where human intervention has a greater impact on land water fluxes^12^. Remote sensing has been widely applied to many studies to analyze the changes of land use and cover and the inter-annual variation of landscape pattern, to assess the ecological security and ecological effect, and to conduct researches about the driving mechanism of such changes^13–24^. Apparently, most studies focus largely on driving mechanisms and ecological effects of land use changes with macro-qualitative or semi-quantitative analysis. There is a lack of in-depth analysis about important roles of water-saving technologies in land use and land cover changes.

Water-saving measures are widely believed to improve utilization efficiency of water resources and further mitigate the crisis in water shortage, especially at an arid area. However, large scale application of water-saving measures may affect hydrological cycles at regional and local scales. Yue *et al*. presented a study about impacts of water-saving measures on groundwater resources at the Hetao Irrigation District in Northwest China where the irrigation-induced infiltration and the groundwater evaporation were the primary factors controlling groundwater table fluctuations during irrigation seasons using the data from 1991 to 2010. The authors reported that conjunctive use of water resources was the most effective way to improve water use efficiency^25^. Berbel *et al*. evaluated the water-saving measures at the Guadalquivir River Basin with the cost-effectiveness analysis and conducted a literature review about linking water savings with water diversion and water depletion from both theoretical models and empirical evidence^26^.

During the last several years, the Manas River Basin (MRB) has become one of the hot topics about impacts of both climate change and human activities on an ecological system because 1) the MRB represents a unique and complex ecological system in an arid area with landscapes ranging from snow cover, mountain, river, plain, oasis, and desert; 2) water availability the main factor that limits reclamation in the MRB; and 3) water-saving measures have been implemented in MRB over 40 years. Feng *et al*. evaluated relationship between the extent of land use change and ecological security grades in the MRB based on the data from 1989–2002 and reported that the ecological security of the drainage basin generally improved somewhat, but deteriorated subsequently in some regions^27^. Liu *et al*. investigated how the water-saving measures such as constructing reservoir in the mountainous region, building water pipes and generalizing water-saving technology in upper, middle and lower reaches can be used to keep sustainable mountain-oasis-ecotone-desert system in the MRB^28^. Zhang *et al*. reported the oases evolution over the last 2,000 years, analyzed the rapid expansion of the Manas River oasis in the last 60 years and examined the relationship between oasis evolution and water resource utilization^29^. Zhang *el al*. evaluated the distribution characteristics of soil salt content in different drip-irrigated soil layers and their effect on the environment in the MRB and reported that extension of the drip-irrigated area has caused a number of environmental problems^30^. However, up to date very few studies have been reported on how to assess specifically water-saving measures on land use and evapotranspiration in the MRB. The main objectives of this study are to characterize the historic change in land use over the period of 1976 to 2015 and temporal and spatial variations in evapotranspiration in the MRB where water-saving measures have been applied over 40 years^31^. We believe that our study is of great significance to enhance our understanding of the interaction mechanism between ecological environments and human activities in arid oasis areas and applicable to other similar areas.

## Description of the Manas River Basin

**Figure 1:**
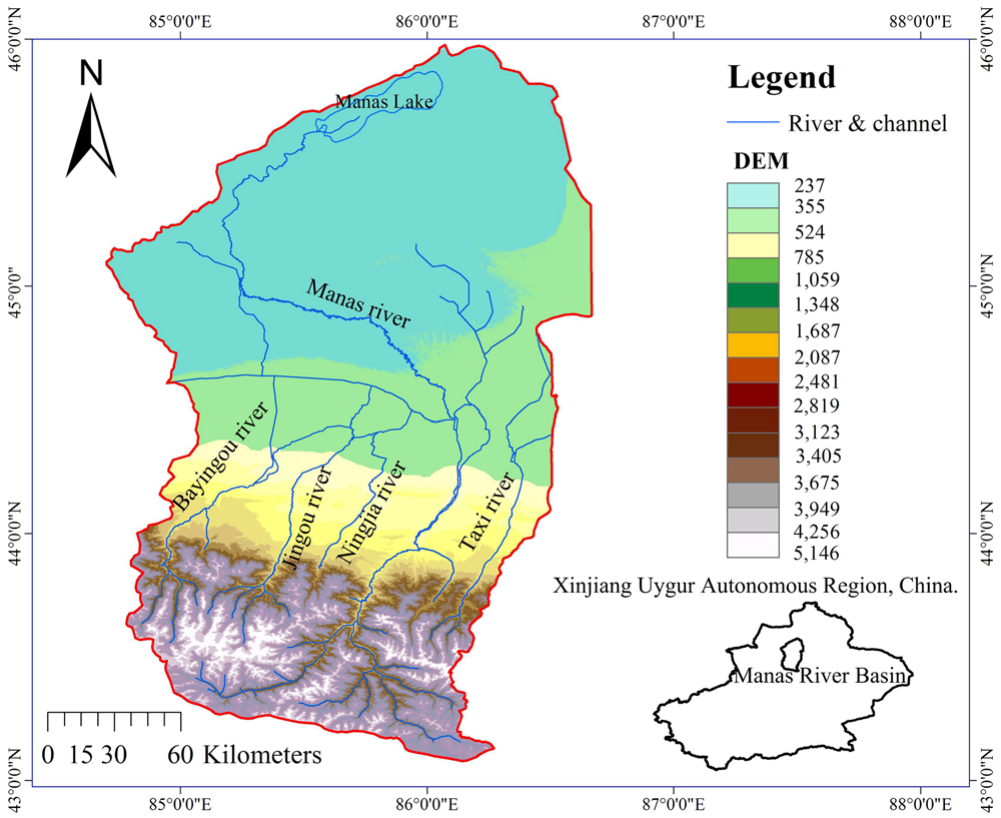
Plots of (**a**) location of the study area and the water transport system, and (**b**) spatial distribution of irrigated at the Manas River Basin^36,37^.

The MRB is located at the center of the North Slope Economic Zone of the Tianshan Mountains, to the southern margin of the Junggar Basin in China (Fig. 1). It is a typical mountain-oasis-desert landscape which can be divided into the upper reach of the mountainous area, the central plain and the lower reach of the desert area^32^. The MRB covers 6 administrative districts, Manas County in ChangJi State, Shawan County in the TaCheng region, Shihezi City, The Eighth Division, the Xinhu area of the Sixth Division, and the Xiaoguai town of Karamay City. At the Manas River Basin, water allocation and utilization is a key factor that determines land use, agricultural production and socio-economic development^32,33^.

Currently, the Manas River Basin is the fourth largest irrigated agricultural area in China. Various water-saving facilities, such as canals, reservoirs, have been constructed to form an excellent water transport system that diverts water from the Manas river to the plains. There are approximately 12 large and medium-sized reservoirs at the central plain with a total reservoir capacity of 5.84 × 10^8^ m^3^. The agricultural ditch system consists of 285 trunk canals and branch canals with a length of approximate 956 km. Approximately 16.4 × 10^8^ m^3^ of water are diverted through the agricultural ditch system to the cultivated lands annually. More than 10 irrigation districts are distributed in the central plain, including the Mosuowan, Shihezi, Xiayedi and Qingshuihe Irrigation Districts^34^. Since the 1960s, areas with agricultural irrigation haven been significantly expanded because of severe shortage in water resources at MRB. More and more groundwater was developed in order to compensate shortage in surface water for agricultural needs^10^.

At the MRB, agricultural irrigation has experienced flood irrigation, ditch irrigation to the development of advanced drip irrigation. After 1996, the film drip irrigation has been widely used at the MRB. The promotion of water saving measures has altered the hydrological cycle at both the local and basin scales. Evapotranspiration is the most important dissipation term, closely related to the ecological environment^33^.

**Figure 2:**
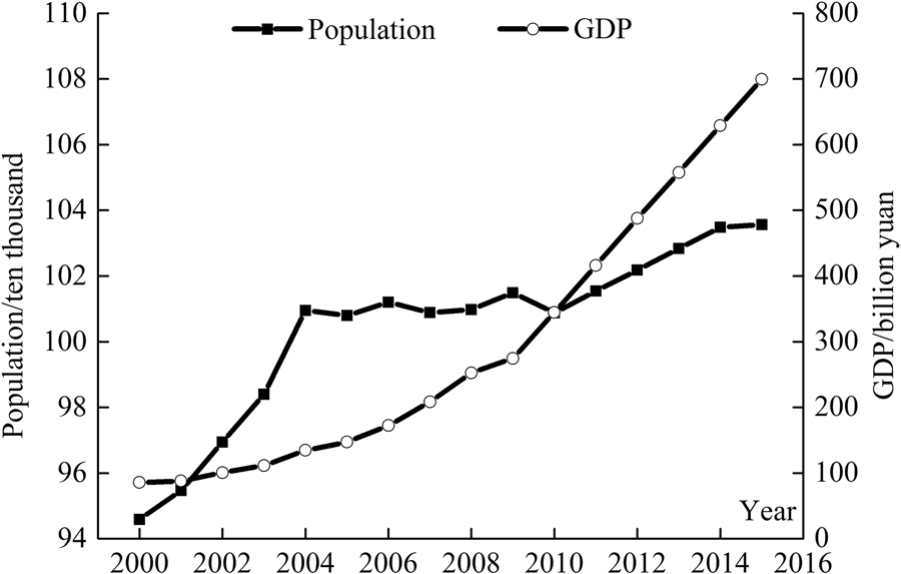
Plot of population and gross domestic production (GDP) over the period of 2000 through 2015 in the Manas River Basin.

Large scale development of water conservation measures, especially agricultural water-saving techniques, makes possible the limited water resources in a basin could meet the needs of population growth and rapid economic development (Fig. 2). The water-saving measures used in the MRB include drip irrigation technology, canal seepage control, dam construction and repair, and other practices. Data in Table 1 show that by the end of 2015 the utilization rate of surface water resources reached 96.27%, and the extraction of groundwater reached almost 56.79%.

**Table 1:** Water resources and utilization in the Manas River Basin, 2015 (Unit: 10^8^ m^3^).

Item	Total	Available	Utilization	the utilization ratio of water resources/%
Surface water	22.91	20.92	20.14	96.27%
Groundwater	11.97	7.43	4.22	56.79%
Springs	/	1.91	1.91	100%
Total	34.88	30.26	26.27	84.35%

With the continuous implementation of drip irrigation technologies, the indices of water saving (water efficiency of canal system, field water utilization coefficient, utilization coefficient of irrigation water) were continuously improved^35^. The water-saving irrigation areas continuously increased (Table 2). By the end of 2015, the water consumption of irrigation was 430 m^3^ha^−1^. The irrigation area with the water-saving measures reached up to 82.1%. Up to date, the water consumption of irrigation is reduced to 400 m^3^ mu^−1^.

**Table 2:** Indices of water saving changes at the Manas River Basin.

Item	1980	2000	2005	2010	2015
Water efficiency of canal system	0.58–0.62	0.65	0.68	0.7	0.76
Field water utilization coefficient	0.61	0.86	0.87	0.89	0.9
Utilization coefficient of irrigation water	0.38	0.57	0.60	0.65	0.68
Canal seepage rate %	10.1	15.4	1.24	24.3	31.3
Water saving irrigation area ratio %	/	4.3	15.6	79.2	82.1
Irrigation water consumption (m³/0.667ha)	/	541	492	449	430

## Research Data

### Data collection and processing

Remote sensing data of land use (i.e., land cover) was derived from the multiple spectral scanner (MSS) images acquired in 1976 and US Terrestrial Satellite TM-ETM images acquired in 1990, 2000, 2010 and 2015 (downloaded from http://glovis.usgs.gov/). In order to take into account the oasis and desertification characteristics at the study area, remote sensing images from August to September each year were selected. Prior to classification of land use, the image preprocessing was standardized, including radiometric calibration, atmospheric correction, and removal of cloud (shadow) pixels. The remote sensing images were clipped within the boundaries of the study area after the mosaic processing. Quality inspection was further conducted to ensure no errors of geometric correction or radiation calibration in the resulting images. The quality inspections were accomplished with the remote sensing classification software eCognition 8.7 (Trimble Inc., Sunnyvale, CA, USA), adopting the Object-Oriented Remote Sensing Feature Information Extraction Method. Then the multi-scale segmentation and classification rules system of TM image were constructed and the relevant feature information of the study area was extracted. The accuracy was evaluated using the confusion matrix method. Various indicators such as the ratio of vegetation coverage to canopy closure (%), coniferous width ratio (%), vegetation height (m), water coverage time of year (month), and the wet index are used to determine the classification system.

MODIS (moderate resolution imaging spectroradiometer) MOD16 global evapotranspiration data (http://www.ntsg.umt.edu/project/mod16) were used as the source of the 2000–2014 evapotranspiration data. The algorithm used to derive the MOD16 surface evapotranspiration data set was an improved algorithm based on the Penman-Monteith formula and surface energy balance theory proposed by Mu *et al*. in 2007 and in 2011. Input data for the MOD16 evapotranspiration data set includes some other MODIS data, such as MOD12Q1 land use data, and MCD43B2 and MCD43B3 albedo data. The non-satellite data, such as the MERRA GMAO (GEOS-5) day-scale meteorological data provided by the United States National Aeronautics and Space Administration, were also used in this study.

The data category, application and temporal and spatial ranges used in the study are listed in Table 3.

**Table 3:** Data used in the research.

Data category	Application	Temporal range	Spatial range
Remote sensing land use data	Analyzing changes in land use	1976, 1990, 2000, 2010, 2015	Path/Row: 144/28; 144/29; 144/30
The remote sensing evapotranspiration data (MOD16A2)	Obtain the evap- otranspiration data set	2000–2014	h23v04; h24v04
Meteorological stations data	Calculate evapotranspiration	2000–2014	
Measured evaporation data	Check MOD16A2 data accuracy	2000–2014	

## Results and Discussion

### Characterization of the land use from 1976 through 2015

**Figure 3:**
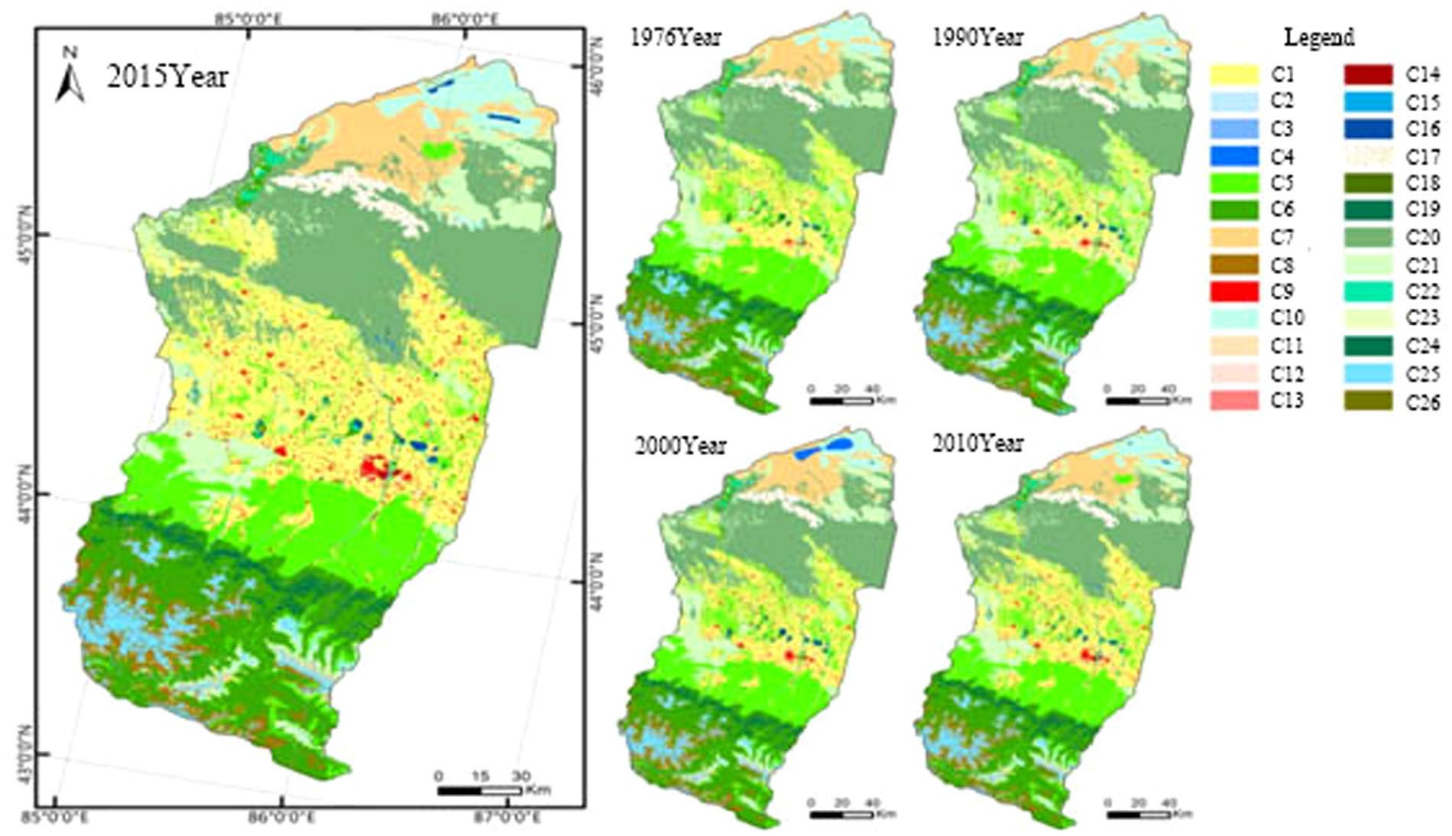
Land use of the Manas River Basin^36,37^. (C1 Dry land; C2 Paddy field; C3 Rivers; C4 Lake; C5 Grassland; C6 Meadow; C7 Bare soil; C8 Bare rock; C9 Residential land; C10 Saline-alkali land; C11 Arbor plot; C12 Arbor greenbelt; C13 Traffic land; C14 Industrial land; C15 Canal; C16 Ponds; C17 Deserts and sandy lands; C18 Shrub land; C19 Evergreen needleleaf forest; C20 Sparse shrub forest; C21 Sparse grassland; C22 Herbaceous swamp; C23 Herbaceous greenbelt; C24 Deciduous broad-leaved forest; C25 Glacier/permanent snow; C26 Deciduous broad-leaved shrub forest).

The total land area of the MRB is about 34,050 km^2^. Figure 3 shows spatial distribution of the current land types in the MRB. Currently, there are 8 types with an area >1000 km^2^, including cultivated land, sparse shrub forest, grassland, meadow, sparse grassland, bare soil, bare rock and glacier in the MRB^36,37^.

In the MRB, the areas of dryland, residential land and meadow were significantly increased over the last 40 years while the areas of the grassland, sparse grassland, sparse shrub and glacier (or permanent snow) land cover exhibited a decreasing trend (Fig. 3). The change in the other land types was relatively minor.

**Figure 4:**
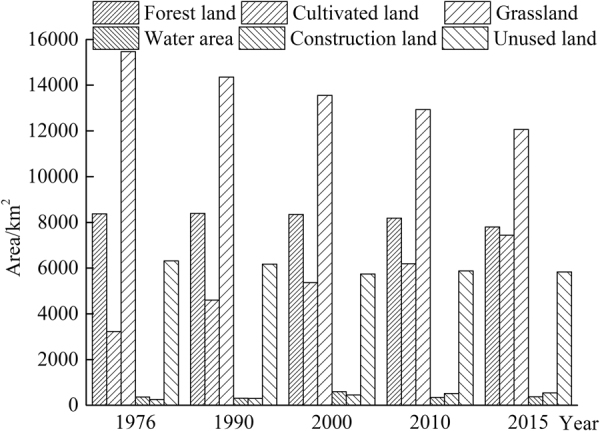
Bar plot of areas of the 6 land types in the Manas River Basin over a period of 1976 to 2015 (Unit: km^2^).

Historic changes in the areas of the 6 major land types (cultivated land, construction land, grassland, forest land, water area and unused land) in the MRB from 1976 through 2015 are shown in Fig. 4. The area of the cultivated land was increased by 130% from 3231 km^2^ in 1976 to 7438 km^2^ in 2015 (Table 4). The area of the construction land was also increased by 118% from 1976 to 2015 (Table 4). Note that the area of the grassland is decreased approximately 22%, from 15,763 km^2^ in 1976 to 12,064 km^2^ in 2015. The forest land and the unused land were decreased modestly by approximately 7% and 8%, respectively. Apparently, the grassland, forest land and water area were changed to the cultivated land with areas of 3744 km^2^, 580 km^2^ and 29 km^2^, respectively over the last 40 years.

**Table 4:** Areas of the 6 major land types in the Manas River Basin over a period of 1976 to 2015 (Unit: km^2^).

	1976	1990	2000	2010	2015
Forest land	8372.30	8392.02	8344.58	8188.10	7799.99
Cultivated land	3230.86	4596.33	5365.43	6187.43	7437.52
Grassland	15463.41	14351.43	13551.33	12938.20	12064.16
Water area	357.69	309.41	593.47	345.51	370.54
Construction land	249.38	298.54	451.78	517.30	544.49
Unused land	6312.90	6169.68	5743.75	5873.80	5833.65

**Figure 5:**
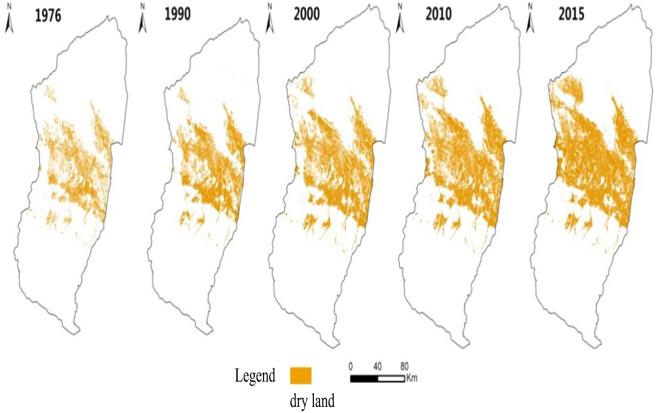
Spatial distribution of the cultivated land in the Manas River Basin in 1976, 1990, 2000, 2010 and 2015^36,37^.

Figure 5 shows change in spatial distribution of the cultivated land in the MRB for the time period of 1976 through 2015. The reclamation rates are estimated at 9.5% in 1976, 13.5% in 1990, 15.8% in 2000, 18.2% in 2010 and 21.8% in 2015, showing an overall increasing trend over the last 40 years. Before 1976, although the local governments implemented aggressive policies to promote land reclamation, the growth of the cultivated land has been primarily limited by the population and the availability of water resources. From 1976 to 1990, due to limitations of the traditional irrigation methods (e.g. flood irrigation), water was not used efficiently in the MRB. As a consequence, secondary soil salinization has been widespread. The cultivated land area was not expanded and the reclamation rate was slowdown. From 1990 to 2015, the cultivated land area increased significantly, largely due to the promotion and application of the film drip irrigation technologies. Consequently, the reclamation rate increased significantly. Table 5 summarizes the four developments of the land use and the water saving measures at the MRB over the time period of 1976 to 2015.

**Table 5:** Analysis of land use for water saving measures at the Manas River Basin, 1976–2015.

Time period	Application of water saving measures	Cultivated land increase (%)	Dynamic degree of cultivated land (%)	Comprehensive index of land use degree
1976–1990	Flood irrigation	42.26	3.0	192.4–197.1
1990–2000	Ditch irrigation	16.73	1.7	197.1–201.5
2000–2010	Drip irrigation	15.32	3.1	201.5–204.0
2010–2015	Film and drip irrigation	20.20	4.0	204.0–207.9

Dynamic degrees of the cultivated land are 3 for 1976–1990, 1.7 for 1990–2000, 3.4 for 2000–2010 and 4.0 for 2010–2015, confirming an overall increase in the cultivated areas in the MRB. Note that after 2000 dynamic degree of the cultivated land is greater than 3. This is likely due to the wide application of drip irrigation in MRB. The comprehensive index of land use in the MRB increased from 192.4 in 1976 to 207.9 in 2015 with an overall increasing trend, suggesting the cultivated land and construction land areas increased while unoccupied land, forest land, grassland and water area decreased. Furthermore, the overall increasing trend in the comprehensive land use degree in the MRB also suggests the increasing degree of human activities on the land use over the time period of 1976 to 2015.

### Characterization of temporal and spatial variations in evapotranspiration

The actual evaporation (ETa) and potential evaporation (ETp) at the MRB were extracted from the MOD16 evapotranspiration vector images and further validated with the observed data collected at the two meteorological stations (Shihezi and Mosowan). Measurements of the actual evapotranspiration at the MRB are very limited. Previous studies show that precipitation can be used to calibrate the actual evapotranspiration at an arid area because the actual evapotranspiration depends upon precipitation. Table 6 lists comparison of the evapotranspiration extracted from the MOD16 and obtained from the two meteorological stations (Shihezi and Mosuwan.) in terms of the root means square error (RMSE), the mean absolute error (MAE) and the average relative error (MRE). Apparently, MAE is highest for the ETp extracted from the MOD16 and measured at the Mosuowan station and the smallest for the Eta extracted from the MOD16 and the measurements at the Shihezi station. The ETa has a smaller MAEs than the ETp does (Table 6). Similarly, the Eta has relatively smaller RMSEs, compared to the ETp. However, the relative errors of ETp extracted from the MOD16 and measured at the two stations are smaller than 5%. The relative errors for the ETa at the two stations are approximately 13%. Apparently, the evapotranspiration extracted from the MOD16 are applicable to the MRB.

**Table 6:** Validation of the actual (ETa) and potential (ETp) evapotranspiration extracted from the MOD16 and observed at the two meteorological stations.

Meteorological station	Shihezi		Mosuowan		
	ETa	ETp	ETa	ETp	
MAE (mm)	28.6	45.8	36.9	58.0	
RMSE (mm)	37.4	57.2	49.3	78.0	
MRE (%)	13.3	4.2	11.3	3.1	

**Figure 6:**
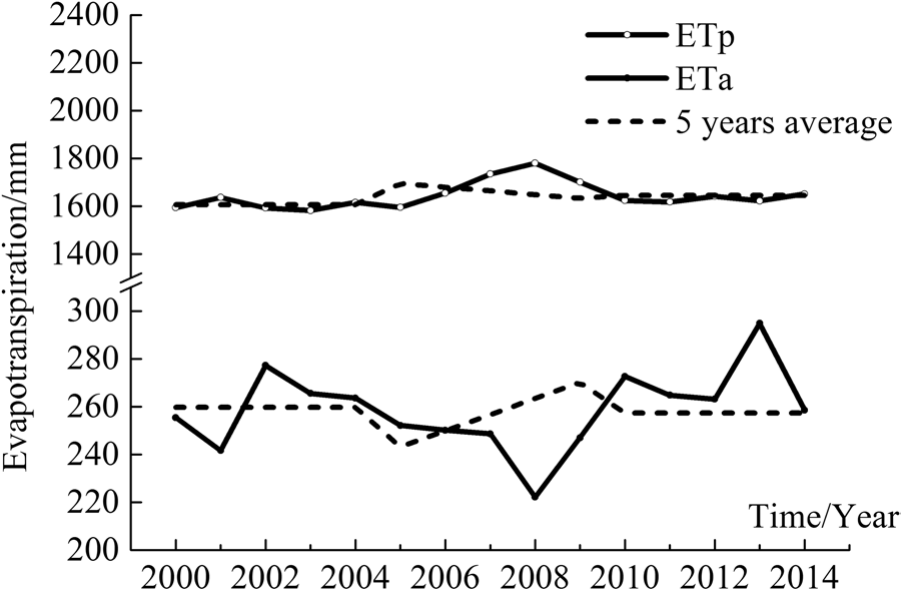
Comparison of the actual (ETa) and potential (ETp) evapotranspiration in the Manas River Basin (MOD16 data).

According to the evapotranspiration extracted from the MOD 16, the average ETa was estimated at 200–250 mm annually and the average ETp was estimated at 1600–1800 mm/year at the basin scale (Fig. 6). The ratio of the annual average ETp to ETa was about 8, indicating the MRB is a typical arid region. To facilitate analysis of trends in ETa and ETp, the evapotranspiration data for 2000–2014 were divided into three periods with 5 years for each (Fig. 6).

During the first period (2000–2004), ETa gradually decreased and ETp gradually increased. In contrast, during the second period (2005–2009) and the third period (2010–2014), the ETa increased while the ETp decreased. Apparently, there was a negative correlation between ETa and ETp in the MRB according to the evapotranspiration data extracted from the MOD 16 (Fig. 6).

**Figure 7:**
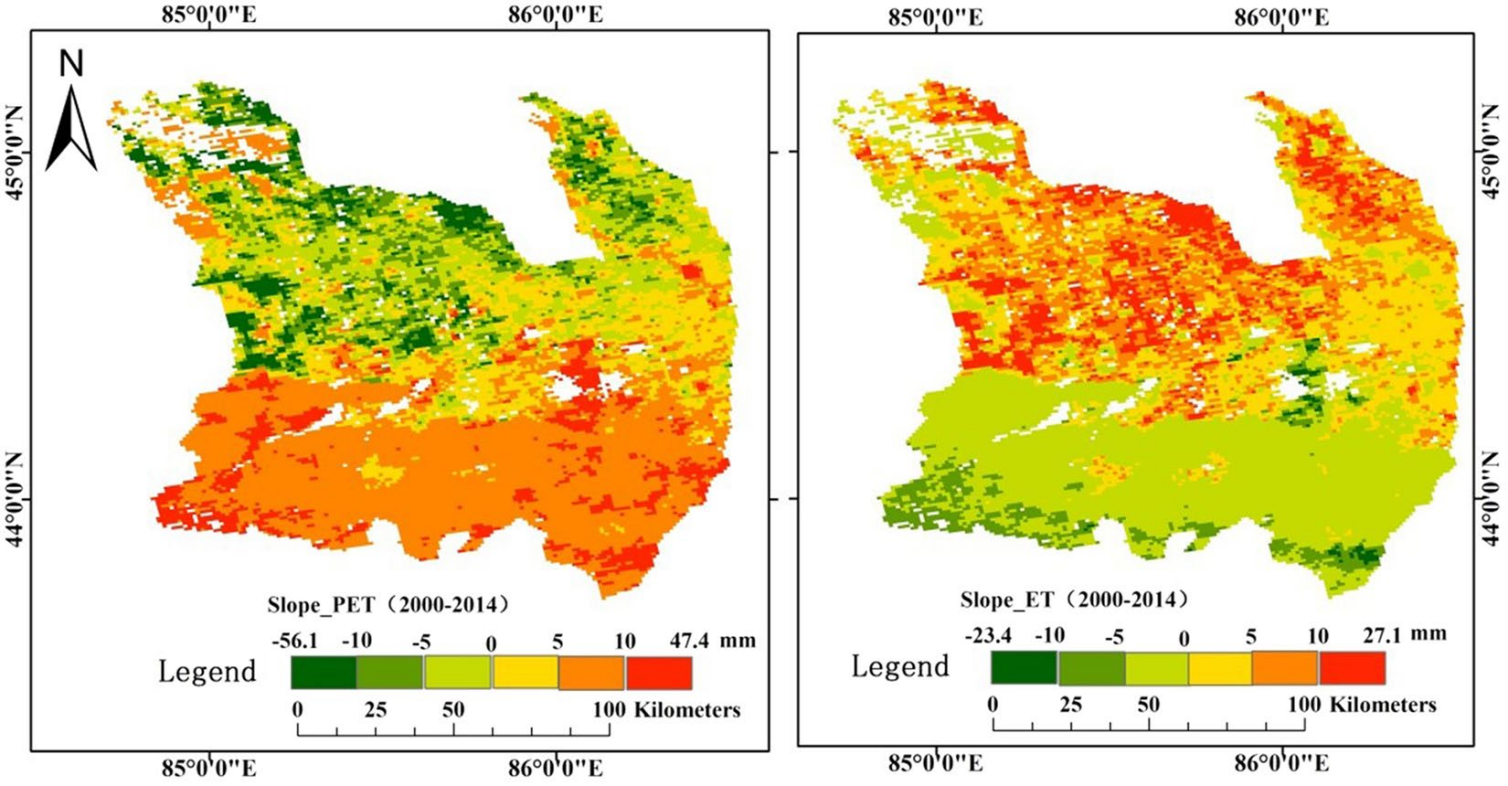
Spatial distribution of the trend slopes estimated for the actual (ETa) and the potential (ETp) evapotranspiration in the Manas River Basin, 2000–2014 with Eq. (4) based on the data extracted from the MOD 16^36,37^.

The overall trends in evapotranspiration in the MRB during 2000–2014 were analyzed with the least square method (Eq.4) and shown in Fig. 7. It can be seen that the ETa and ETp followed an opposite trend in their spatial variations (Fig. 7). The ETa increases at the cultivated lands in the central plain area, but decreases in all other land areas. In contrast, ETp decreases at the cultivated land of the central plain, but increases elsewhere at the MRB. This suggests that the cultivated land at the plain areas of the MRB is more humid than the other areas. The reason that ETa showed an increasing trend at the areas with the human activities and a decreasing trend in other regions is likely due to change in the land surface conditions caused by the promotion of water-saving measures that affect the type of land use and further alter the water cycle process.

The area with an increasing trend in the ETa is about 16.6% of the total area of the MRB, mainly at the cultivated land (Fig. 7). The areas with a decreasing trend in the ETa are mainly distributed in the grassland and forest area in the southern mountain area. The area with an increasing trend of the ETp is about 79.3% of the total area of the MRB, distributed in the regions except the plain areas. The areas with a decreasing trend in ETp are mainly distributed in the plain area at the MRB (Fig. 7).

The actual evapotranspiration of the basin shows an increasing trend in the central plain, while the remaining areas show a decreasing trend during the 2000–2014 years of water-saving technology popularization. The potential evapotranspiration shows a decreasing trend in the central plain, while the remaining areas show an increasing trend. It shows that the plain area in the middle reaches of the Manas River Basin is becoming more and more humid, while the other regions show more and more drought, and the oasis and desertification in the basin are aggravating simultaneously under the function of water-saving technology.

Manasi River Basin has been developed into the largest oasis farming area in Xinjiang and the fourth largest irrigation agriculture area in China after more than 20 years of water saving technology popularization and application. Almost northwest area of China is mountain and basin system, water resources originated in the mountains, and utilization in the oasis area, dissipated in desert, therefore, the research methods has reference significance in land use and evapotranspiration research for arid area of Northwest Chinas especially with strong human activities.

## Concluding Remarks

In this study the land use over a period of 1976–2015 and temporal and spatial distributions of evapotranspiration over a period of 2000–2014 at the Manas River Basin, China were assessed with the remote sensing and geographic information system analysis. Impacts of the water-saving measures on changes in land use types and the temporal and spatial variations of evapotranspiration in an arid oasis area were analyzed quantitatively. Our results show that water-saving measures in agriculture promoted expansion in the cultivated land and further resulted in change in the land use over the period of 1976–2015. On the other hand, the cultivated land expansion led to degradation in the grass land, with aggravation of oasis and desertification. Furthermore, this study also shows that the crops coverage increased, natural vegetation decreased and desertification area expanded at the MRB over the period of 1976–2015.

Spatial and temporal patterns in evapotranspiration at the MRB were characterized in the MRB over the period of 2000–2014 that water-saving measures promoted agricultural activities. Estimation of the actual evapotranspiration and the potential evapotranspiration suggests that the actual evapotranspiration reached its minimum value and the potential evapotranspiration was the highest in 2008 in the MRB. As a consequence, the MRB was the driest in 2008 over the period of 2000–2014. At the central plain area, the cultivated land tended to be wetter, while the other regions experienced more droughts. This study suggests that water-saving measures changed the long-term hydrological cycle in MRB through altering evapotranspiration. The methods presented in this study can be applied to other arid areas in the world. In addition, the results of this study provide scientific guidance for policy makers to regulate water resource allocation and utilization in an arid area.

## Methods

### Analysis of the land use

Three indicators (reclamation rate, dynamic degree of single land use and comprehensive index of land use degree) were used to evaluate change in the land use in MRB over the last 40 years.

1. Reclamation rate, *P*, is defined as a percentage of the cultivated land area (A_C_) to the total area (A_T_) of the MRB,
  1 $${\rm{P}}={A}_{C}/{A}_{T}\times 100 \% $$
2. Dynamic degree of single land use (*K*), describing change in use of a specific land type at the study area for a particular time period, is given by Eq. (2),
  2 $$K=({U}_{b}-{U}_{a})/{U}_{a}\times \frac{1}{T}\times 100 \% $$
  where *U*_*a*_ and *U*_*b*_ are areas of land use for a particular land type at the beginning and the end of the time period, respectively; and *T* is the length of the time period (years). *K* represents the annual rate of relative change in land use. Note that a positive *K* indicates an increasing trend for this particular land type whereas a negative *K* suggests a decreasing trend.
3. Comprehensive index of land use degree (L), an indicator of change in land use degree, is defined as the following equation,

3 $$L=100\sum _{m=1}^{n}(C\times {P}_{m})$$

where C takes a value of 1 for the unused land, 2 for the woodland, grass land and water, 3 for cultivated land, and 4 for the construction land, *P*_*m*_ is the area percentage for each of the four categories in the MRB and *n* is equal to 4. The comprehensive index of land use degree, *L*, has a value, ranging from 100 to 400. Note that *L* indicates both the land use degree and human interventions on the land use.

### Analysis of evapotranspiration

The overall trend of evapotranspiration in MRB over the period of 2000–2014, *S*, was determined using the least square method based on the following equation,

4 $$S=\frac{{\sum }_{{\rm{i}}=1}^{{\rm{n}}}({\rm{i}}\times {{\rm{ET}}}_{{\rm{Mi}}})-({\sum }_{{\rm{i}}=1}^{{\rm{n}}}{\rm{i}})({\sum }_{{\rm{i}}=1}^{{\rm{n}}}{{\rm{ET}}}_{{\rm{Mi}}})/{\rm{n}}}{{\sum }_{{\rm{i}}=1}^{{\rm{n}}}{{\rm{i}}}^{2}-{({\sum }_{{\rm{i}}=1}^{{\rm{n}}}{{\rm{ET}}}_{{\rm{Mi}}})}^{2}/{\rm{n}}}$$

where *n* ( = 15) is the length of the time period and ET_*mi*_ is the evapotranspiration (actual ET or potential ET) extracted from the MOD16 dataset at the i^th^ year. A negative value of *S* indicates the change trend of evapotranspiration is decreasing. Otherwise, evapotranspiration has an increasing trend over the period of 2000 to 2014.

### Data availability statement

We declare the availability of the data in our manuscript.
